# Population growth lags in introduced species

**DOI:** 10.1002/ece3.7352

**Published:** 2021-03-09

**Authors:** Catherine L. Kelly, Lin Schwarzkopf, Iain J. Gordon, Ben Hirsch

**Affiliations:** ^1^ Division of Tropical Environments and Societies James Cook University Townsville Qld Australia; ^2^ Fenner School of Environment & Society Australian National University Canberra ACT Australia; ^3^ James Hutton Institute Aberdeen UK; ^4^ Australian Tropical Science and Innovation Precinct CSIRO Townsville Qld Australia; ^5^ Central Queensland University Townsville Qld Australia; ^6^ Smithsonian Tropical Research Institute Panama City Panama

**Keywords:** invasive species, lag phase, population growth, prolonged lag, ungulates

## Abstract

When introduced to new ecosystems, species' populations often grow immediately postrelease. Some introduced species, however, maintain a low population size for years or decades before sudden, rapid population growth is observed. Because exponential population growth always starts slowly, it can be difficult to distinguish species experiencing the early phases of slow exponential population growth (inherent lags) from those with actively delayed growth rates (prolonged lags). Introduced ungulates provide an excellent system in which to examine lags, because some introduced ungulate populations have demonstrated rapid population growth immediately postintroduction, while others have not. Using studies from the literature, we investigated which exotic ungulate species and populations (*n* = 36) showed prolonged population growth lags by comparing the doubling time of real ungulate populations to those predicted from exponential growth models for theoretical populations. Having identified the specific populations that displayed prolonged lags, we examined the impacts of several environmental and biological variables likely to influence the length of lag period. We found that seventeen populations (47%) showed significant prolonged population growth lags. We could not, however, determine the specific factors that contributed to the length of these lag phases, suggesting that these ungulate populations' growth is idiosyncratic and difficult to predict. Introduced species that exhibit delayed growth should be closely monitored by managers, who must be proactive in controlling their growth to minimize the impact such populations may have on their environment.

## Abstract 2

Our study uses ungulates as a model system to investigate how environmental factors affect the growth of introduced species. As many introduced ungulates are conservation threats, understanding factors that influence their growth is important for effective management and control strategies. We found that of 37 ungulate populations around the world, 16 showed significant lags in population growth although the causes for these lags could not be identified, and are presumed to be idiosyncratic.

## 1 INTRODUCTION

Non‐native species have been introduced worldwide and often demonstrate rapid population growth postrelease (Chollet et al., 2015; Froese et al., 2017; Ikagawa, 2013). Many introduced species threaten their introduced environments by competing directly and indirectly with native species for food and water (Dolman & Waber, 2008; Witte et al., 2010), consuming native species (Angel et al., 2009; Cole & Litton, 2014; Innes et al., 2010; Kardol et al., 2014), and spreading disease (Crowl et al., 2008; Strickland et al., 2015).

After a species is introduced to a non‐native environment, its population may increase rapidly. Even in these species, a time delay between the introduction of a species to a new area and rapid population growth normally exists (Binggeli, 2001; Kowarik, 1995). Crooks and Soule (1999) defined this delay as an ‘inherent lag,’ or the normal early period of exponential growth that occurs before the inflection point in the curve of population growth. Prolonged lag phases occur when a species persists in an environment in low numbers for an extended period (Daehler, 2009; Rilov et al., 2004). There are two basic types of lags: lags in population growth and lags in population spread, although the two can appear synonymous (Crooks & Soule, 1999). To determine whether a lag is ‘prolonged’ (i.e., an extended period of even slower growth than that predicted from exponential growth, which occurs prior to a marked increase in the rate of growthAagaard & Lockwood, 2014; Crooks, 2005), it is necessary to determine whether the observed lag is longer than the inherent lag. We follow these definitions and statistically distinguish between natural exponential population growth and prolonged lags (Aikio et al., 2010; Crooks, 2005; Crooks & Soule, 1999; Sakai et al., 2001) for ungulate species introduced into non‐native ranges.

Prolonged lags can be distinguished using models of expected growth, which can be compared with the observed pattern of growth. If the observed rate of population growth is much lower than predicted in the expected growth model, then the population is experiencing a prolonged lag (Crooks, 2005; Hengeveld, 1989). Although the idea that a population might experience lag phases longer than those dictated by exponential growth has been recognized for many years, most studies have not tested whether prolonged lags (sensu stricto) have occurred (cf., Aagaard & Lockwood, 2014; Aikio et al., 2010). Additionally, previous studies that have investigated prolonged lags have focused primarily on invertebrate and plant populations, with little attention given to introduce vertebrates (cf., Aagaard & Lockwood, 2014).

A number of factors may influence the length of lag phases in population growth, such as changes in environmental conditions, behavioral plasticity, genetic adaptation, or changes in interactions between the invading species and their surrounding environment (Crooks & Soule, 1999; Rilov et al., 2004; Wang & Wang, 2006; Witte et al., 2010). There are also lags in the detection of invasive species that can provide incorrect information on initial population growth (e.g., invaders could be present in low numbers before first being detected; Crooks & Soule, 1999). Any one, or all of these factors, may impact the population growth of invasive species to different degrees, although this appears to be largely unpredictable (Mack et al., 2000). For example, croton weed (*Areratina adenophora*) is invasive in China and exhibited a lag phase of 20 years before suddenly expanding throughout southern China, potentially due to favorable environmental conditions (Wang & Wang, 2006). In invaded regions with favorable conditions for growth, *A. adenophora* has expanded its range at a rate of 20 km/year. In contrast, in less favorable areas, it expanded much more slowly (3.7 km–9.8 km/year). Environmental factors such as rainfall and temperature may also have influenced range expansion both positively and negatively in different areas (Wang & Wang, 2006), but little is known about the influence of the environment on the initial population growth of introduced species.

The factors that cause prolonged lag phases, or a species' release from them, may be idiosyncratic and vary among species and populations (Aagaard & Lockwood, 2014; Larkin, 2012). A wide range of changing interactions between invading species and the surrounding biotic and abiotic environment could potentially enhance the fitness of an invasive species, triggering an increase in its population growth (Crooks & Soule, 1999; Rilov et al., 2004; Witte et al., 2010). A species could also increase rapidly following the emergence of new mutations and genotypes more suitable to the new environment (Crooks, 2005; Mack et al., 2000). Similarly, an expanding invasive species that suddenly experiences favorable environments may exhibit sudden, rapid population growth, after a period of prolonged lag (Crooks, 2005). The Eurasian collared dove (*Streptopelia decaocto*) spreading through Syria and Turkey in the 16th century exhibited a lag phase of approximately 200 years (Crooks & Soule, 1999). Its sudden range expansion was attributed to changes in climate, and to an increase in the availability of human‐altered environments (Crooks & Soule, 1999; Fujisaki et al., 2010; Romagosa & Labisky, 2000). Similarly, the fire‐adapted heath banksia (*Banksia ericafolia*), introduced to South Africa, remained in a lag phase for over 40 years until several fires caused a sudden and rapid increase in its abundance (Geerts et al., 2013).

Understanding a population's initial growth phase and trying to clarify potential causes of prolonged lags is critical for designing and implementing management strategies for extermination. To determine which populations lagged for periods longer than the inherent lag phase, and to discern which factors may cause prolonged lags in population growth, we used ungulates as a model system. Ungulates have been widely introduced to environments around the world, primarily as a game or food resources, and can be extremely damaging as invasive species (Cote et al., 2004; Forsyth & Hickling, 1998; Hernandez et al., 2018; Ikagawa, 2013; Riney, 1964). Their well‐documented introduced populations provide an opportunity to investigate rates of growth postrelease in several environments and conditions. Typically, introduced ungulate populations increase rapidly postliberation, usually exceeding their carrying capacity, after which their populations crash (e.g., reindeer *Rangifer* sp.; Scheffer, 1951; Riney, 1964; Klein, 1968). Most population studies of ungulates have focused on investigating the factors causing the crash phase of population growth, while the initial growth phase is often neglected (Forsyth & Caley, 2006; Kaji et al., 2004; Riney, 1964).

In this study, we used abundance data to (a) distinguish inherent from prolonged lags in published studies of the population growth of introduced ungulates and (b) determine which environmental or life‐history factors might have affected the length of the lag phase, or if lag phases are idiosyncratic (i.e., that the factors that influence population growth varied among species and populations). We selected a range of environmental variables that may have contributed to the population growth of introduced species. This included rainfall metrics (total annual rainfall, intra‐annual rainfall variance, and the length of the dry season), location information (temperate or tropical environment, island or mainland environment, average annual temperature), and other factors including gestation period, presence of interspecific relationships (predators or competitors), and if the population was reported to be subject to hunting pressure. While all these variables may influence population growth, we thought several would contribute to the occurrence and length of prolonged lags. Considering the environmental conditions that typically reduce reproductive output and mortality in ungulates, we predicted that species with long gestation periods living in areas with more competitors and longer dry seasons would be more likely to exhibit prolonged lags (Coe et al., 1976; Fryxell et al., 1988; Garel et al., 2004). In contrast, we expected species with shorter gestation periods, introduced to areas with no native competitors, and consistent rainfall throughout the year would exhibit earlier, faster population growth.

## 2 METHODS

We used data from 33 published studies of 25 introduced ungulate species, and 36 populations (Table 1). To locate relevant literature, we searched the James Cook University library digital database (https://www.jcu.edu.au/library) and Google Scholar (https://scholar.google.com/) using the following terms: “introduced,” “invasive,” “feral,” “non‐native,” “exotic,” “population,” and/or “introduction” followed by “ungulate” or a family name such as “cervid” or “deer.” The reference lists in each publication were checked for any additional publications not on our list. In some cases, publications alluded to, but did not specifically reference an introduced population. In these cases, a specific search was performed. Populations of liberated domestic species were excluded. Studies were retained for analysis if they included information on the founding population size, an estimate of the period of population growth, and a population size at a later date. Studies were also only included when they described a novel introduction or colonization. Populations that were reintroductions to previously inhabited areas were excluded. Many of the studies included here (*n* = 28) only had two population estimates: the initial size of the founding population and the population size at one point later in time. Populations in this analysis were isolated from other introduced populations so counts could not have been influenced by immigration or emigration. We determined gestation period, age at sexual maturity, maximum number of offspring produced per year, and average maximum age (i.e., adult survival) in the wild for each species for which population data were available (Table 1). Where available, we used data specific to each introduced population (i.e., the same data as listed above but specific to each introduced population). In some cases (*n* = 21), there were insufficient life‐history data available for the introduced population, so information from another population in the species' native range was used.

**TABLE 1:** List of species included in this study

Species (source)	Location	Growth Period	Initial number	Surveyed number	Weather data source	Life‐history sources
*Rangifer tarandus* (St Matthew) (1)	Alaska	19	29	6,000	NOAA—US Department of Commerce	Gunn (2016)
*Rangifer tarandus* (Grande‐Terre) (2)	Kerguelen Isl.	16	7	2,000	MET Office	Gunn (2016)
*Ovis gmelini musimon* (2)	Kerguelen Isl.	12	2	100	MET Office	Santiago‐Moreno et al. (2005)
*Rangifer tarandus* (St George) (3)	Alaska	11	15	222	NOAA—US Department of Commerce	Gunn (2016)
*Rangifer tarandus* (Haute) (2)	Kerguelen Isl.	15	3	115	MET Office	Gunn (2016)
*Antilocapra americana* (4)	Hawaiꞌi	7	38	250	NOAA—US Department of Commerce	Tomich (1969)
*Odocoileus hemionus* (5)	USA	17	22	2,000	NOAA—US Department of Commerce	Robinette et al. (1973)
*Ovis Canadensis* (6)	Mexico	18	16	700	CICESE	Festa‐Bianchet (2008)
*Capreolus capreolus* (7)	Germany	20	8	550	Deutscher Wetterdienst	Hoffmann et al. (1978)
*Cervus nippon* (8)	Japan	12	54	592	Japan Meteorological Agency	McCullough et al. (2009)
*Cervus timorensis* (9)	Australia	21	7	850	Bureau of Meteorology	Hedges et al. (2008)
*Cervus unicolor* (10)	New Zealand	24	2	100	The National Climate Database‐NIWA	King (2005)
*Odocoileus virginianus* (11)	Finland	27	5	1,000	Finnish Meteorological Institute	Kekkonen et al. (2016)
*Ammotragus lervia* (12)	Spain	19	34	2,000	Murcia MET	Abaigar et al. (2012)
*Rangifer tarandus* (St Paul) (3)	Alaska	27	25	1,943	NOAA—US Department of Commerce	Gunn (2016)
*Ammotragus lervia* (13)	USA	10	85	500	NOAA—US Department of Commerce	Abaigar et al. (2012)
*Ovis gmelini* (14)	Hawaiꞌi	30	11	2,500	NOAA—US Department of Commerce	Tomich (1969)
*Dama dama* (15)	USA	20	28	550	NOAA—US Department of Commerce	Asher et al. (1988)
*Ovis gmelini* (16)	Canary Isl.	22	11	400	Murcia MET	Garel et al. (2005)
*Lama guanicoe* (17)	Falkland Isl.	17	15	275	Climate Research Unit	Riveros et al. (2015)
*Oryx gazella gazelle* (18)	USA	24	95	3,500	NOAA—US Department of Commerce	Dieckmann (1980)
*Ammotragus lervia* (16)	Canary Isl.	18	16	250	Murcia MET	Abaigar et al. (2012)
*Rangifer tarandus* (19)	South Georgia Isl.	46	10	3,000	World Weather Online	Gunn (2016)
*Rusa timorensis* (20)	New Caledonia	70	12	200,000	World Weather Online	Leslie (2011)
*Cervus nippon* (21)	USA	42	5	300	NOAA—US Department of Commerce	McCullough et al. (2009)
*Axis axis* (15)	USA	28	36	461	NOAA—US Department of Commerce	Graf and Nichols (1966)
*Hemitragus jemlahicus* (22,23)	New Zealand	46	21	710	The National Climate Database ‐NIWA	King (2005)
*Axis axis* (24,25)	Australia	130	4	44,000	Bureau of Meteorology	Chapple (1989)
*Axis axis* (26)	Hawaiꞌi	98	8	6,000	NOAA—US Department of Commerce	Graf and Nichols (1966)
*Oreamnos americanus* (27)	USA	44	170	2,355	NOAA—US Department of Commerce	Lauer et al. (1999)
*Bubalus bubalis* (28)	Australia	142	80	340,000	Bureau of Meteorology	Boulton and Freeland (1991)
*Camelus dromedarius* (29)	Australia	84	4,500	600,000	Bureau of Meteorology	Pople and McLeod (2010)
*Odocoileus virginianus* (30)	Canada	120	220	160,000	Government of Canada	Kekkonen et al. (2016)
*Hydropotes inermis* (31)	England	96	19	4,000	MET Office	Dubost et al. (2011)
*Bos javanicus* (32)	Australia	158	20	10,000	Bureau of Meteorology	Bradshaw and Brook (2007)
*Cervus nippon* (33)	Poland	29	54	121	TuTiempo	McCullough et al. (2009)

Note

Growth period represents the period of time between the initial introduction and the surveyed number (largest population recorded in the literature).

*Sources:* (1) Klein (1968); (2) Chapui et al. (1994); (3) Scheffer (1951); (4) Tomich (1969); (5) Dvorak and Catalano (2016); (6) Colchero et al. (2009); (7) Steinbach et al. (2018); (8) Kaji et al. (2004); (9) Webley et al. (2004); (10) Thomson (1922); (11) Kekkonen et al. (2012); (12) Cassinello et al. (2004); (13) Cassinello (1998); (14) Judge et al. (2017); (15) Gogan et al. (2001); (16) Nogales et al. (2006); (17) Franklin and Grigione (2005); (18) Bender et al. (2019); (19) Leader‐Williams (1980); (20) Barrau and Devambez (1957); (21) McCullough et al. (2009); (22) Caughley (1970); (23) Tustin and Challies (1978); (24) Bentley (1967); (25) Brennan and Pople (2016); (26) Graf and Nichols (1966); (27) Flesch et al. (2016); (28) Boulton and Freeland (1991); (29) Saalfeld and Edwards (2010); (30) Fuller et al. (2018); (31) Cooke (2009); (32) Bradshaw and Brook (2007); (33) Kopij (2017).

To create a model of population increase for unrestricted growth, exponential models were generated using the founding population size, average maximum age in the wild, average number of offspring produced by each female per year, average age at sexual maturity, and assuming equal sex ratios. It was these models that were used to calculate the population sizes under exponential growth, and later used to calculate the doubling time assuming exponential growth.

We compared empirical measures of population growth rates with our estimates of maximum possible exponential growth rates. Empirical population growth was determined using the initial and final population sizes found in our literature search (Table 1). 95% confidence intervals were then calculated for the empirical growth curve and populations were classified as having exhibited a prolonged lag phase when the slope of a population's exponential curve fell outside 95% confidence intervals of the empirical model (using the method suggested by Crooks & Soule, 1999). We used a binary response variable of lag (1) or nonlag (0) to investigate the factors that influenced the likelihood of a population exhibiting a prolonged lag in the statistical models described below.

To estimate the growth rates of these species, we calculated the doubling time using the following formula (Hulting et al., 1990):

dt=log(2)/r

where *r* is the annual rate of increase (∆population/∆time). Delta time, as used here, was the same for both empirical and exponential populations. The difference in doubling time between the empirical and exponential models (calculated from population growth in the exponential models) was then calculated and used as a response variable, also described below Table 2).

**TABLE 2:** Doubling time (DT) of introduced ungulates compared with exponential population models, sorted in descending order from lowest to highest difference in doubling time

Species (source)	Location	Introduced environment	Observed DT	Exponential DT	Difference
*Rangifer tarandus* (St Matthew) (1)	Alaska	Nontropical	2.47	2.40	0.07
*Rangifer tarandus* (Grande‐Terre) (2)	Kerguelen Isl.	Nontropical	1.96	1.82	0.14
*Ovis gmelini musimon* (2)	Kerguelen Isl.	Nontropical	2.13	1.87	0.26
*Rangifer tarandus* (St George) (3)	Alaska	Nontropical	2.83	2.50	0.33
*Rangifer tarandus* (Haute) (2)	Kerguelen Isl.	Nontropical	2.85	2.41	0.44
*Antilocapra americana* (4)	Hawaiꞌi	Tropical	2.84	2.33	0.50
*Odocoileus hemionus* (5)	USA	Nontropical	2.61	1.83	0.78
*Ovis Canadensis* (6)	Mexico	Nontropical	3.30	2.45	0.85
*Capreolus capreolis* (7)	Germany	Nontropical	3.28	2.42	0.86
*Cervus nippon* (8)	Japan	Nontropical	3.47	2.43	1.04
*Cervus timorensis* (9)	Australia	Nontropical	3.03	1.73	1.30
*Cervus unicolor* (10)	New Zealand	Nontropical	4.25	2.93	1.32
*Odocoileus virginianus* (11)	Finland	Nontropical	3.53	1.80	1.73
*Ammotragus lervia* (12)	Spain	Nontropical	3.23	1.46	1.77
*Rangifer tarandus* (St Paul) (3)	Alaska	Nontropical	4.30	2.50	1.80
***Ammotragus lervia* (13)** ^a^	**USA**	**Nontropical**	**3.91**	**1.90**	**2.01**
*Ovis gmelini* (14)	Hawaiꞌi	Tropical	3.83	1.80	2.03
*Dama dama* (15)	USA	Nontropical	4.66	2.40	2.26
*Ovis gmelini* (16)	Canary Isl.	Nontropical	4.24	1.82	2.42
***Lama guanicoe* (17)** ^a^	**Falkland Islands**	**Nontropical**	**4.05**	**1.49**	**2.56**
***Oryx gazella gazelle* (18)** ^a^	**USA**	**Nontropical**	**4.61**	**1.97**	**2.64**
*Ammotragus lervia* (16)	Canary Isl.	Nontropical	4.54	1.84	2.70
***Rangifer tarandus* (19)** ^a^	**South Georgia Isl**.	**Nontropical**	**5.59**	**2.37**	**3.22**
***Rusa timorensis* (20)** ^a^	**New Caledonia**	**Tropical**	**5.35**	**2.35**	**3.00**
***Cervus nippon* (21)** ^a^	**USA**	**Nontropical**	**7.11**	**2.33**	**4.78**
***Axis axis* (15)** ^a^	**USA**	**Nontropical**	**7.61**	**2.39**	**5.22**
***Hemitragus jemlahicus* (22,23)** ^a^	**New Zealand**	**Nontropical**	**9.06**	**2.32**	**6.74**
***Axis axis* (24,25)** ^a^	**Australia**	**Tropical**	**9.69**	**2.35**	**7.34**
***Axis axis* (26)** ^a^	**Hawaiꞌi**	**Tropical**	**10.26**	**2.34**	**7.92**
***Oreamnos americanus* (27)** ^a^	**USA**	**Nontropical**	**11.60**	**2.98**	**8.63**
***Bubalus bubalis* (28)** ^a^	**Australia**	**Tropical**	**11.78**	**2.77**	**9.01**
***Camelus dromedarius* (29)** ^a^	**Australia**	**Tropical**	**11.90**	**2.77**	**9.13**
***Odocoileus virginianus* (30)** ^a^	**Canada**	**Nontropical**	**12.62**	**1.81**	**10.82**
***Hydropotes inermis* (31)** ^a^	**England**	**Nontropical**	**12.44**	**1.52**	**10.92**
***Bos javanicus* (32)** ^a^	**Australia**	**Tropical**	**17.62**	**2.87**	**14.75**
***Cervus nippon* (33)** ^a^	**Poland**	**Nontropical**	**24.91**	**2.37**	**22.54**

^a^ Species for which the theoretical exponential growth rate was significantly less than the 95% confidence intervals of empirical exponential growth models. Bold was supplementary to asterisk, they both indicate species for which the theoretical exponential growth rate was significantly less than the 95% confidence intervals of empirical exponential growth models.

After determining which populations showed significant prolonged lags (*n* = 17), we examined possible causes of variation in population growth rates using generalized linear models, and a range of different explanatory variables associated with climate and life history (Table 3). To determine the degree of lag shown by each lagging population, we calculated the difference between the theoretical population growth rate calculated for each lagging population, and its actual growth rate, and used this difference as our response variable in the models. One disadvantage of this method is that it was not possible to detect population crashes or changes in growth rate between the starting and ending points. In our dataset, there were only eight species with more than two available population estimates. We found that the theoretical growth trajectories in these eight species were largely similar to the empirical data (see Supplementary Material 1) and thus concluded that our estimation method reflected biological reality reasonably well.

**TABLE 3:** Description of variables derived from meteorological databases and primary literature to select candidate models for population growth of introduced ungulates

Variable Name	Variable Description
Dry season	The number of consecutive months with rainfall in the lowest 25% of monthly rainfall, correlating with the length of the dry season
Gestation	The gestation period of each species (days)
Region	A categorical variable indicating if the population was introduced to a tropical or temperate environment
Rainfall	The average annual rainfall (mm)
Temperature	The average annual temperature (°C)
Island	A categorical variable indicating if the population was introduced to an island or the mainland
Predators	A categorical variable indicating if the population was introduced to a location with potential predators
Competition	A categorical variable indicating if the population was introduced to an area with native competitors
Hunting	A categorical variable indicating whether the population was introduced for hunting purposes
Variance	Average intra‐annual rainfall variance

Climate data were obtained from meteorological databases and public weather stations (including temperature and historic monthly rainfall from weather stations as close as possible to the release site of each population; Table 1). The climatic variables for each location included mean annual rainfall (mm) and temperature (°C) (calculated from the entire available weather station dataset). The number of consecutive months with rainfall in the lowest 25% per annum was calculated to estimate the length of the dry season (i.e., the lowest 25% monthly rainfall measurements available from the entire dataset available from the appropriate weather station). A categorical variable: “island” or “mainland” was also included. The gestation period of each species was also determined from the literature and used as an explanatory variable in the model. Other intrinsic variables (e.g., maximum age in wild, age at sexual maturity, etc.) were used to generate models for population growth and, therefore, were not included here. In addition, we classified whether populations were introduced to areas with native natural predators or competitors. A species list was obtained for each area and if there was at least one native potential competitor (mega‐herbivore) or predator (medium‐to‐large carnivore), that population was said to have had potential competition or predation pressure. Finally, we also included if the literature suggested a population was subject to hunting. To account for effects potentially caused by using the same species more than once in the model and among‐species differences, species was included as a random effect in the model. We also conducted all analyses with various calculated measures of growth as response rates (annual rate of increase (*r*) and the slopes of the curves; Supplementary Material 2).

All analyses were conducted in R (V3.4.1, R Studio Team, 2017) and visualized using the *ggplot2* package (Wickham, 2016). We performed model selection based on Akaike's information criterion (AICc) to select the best subset models of population growth. Due to multicollinearity, island, total annual rainfall, and temperature were removed from model selection. We tested all combinations of gestation period, months of consecutive low rainfall, average annual rainfall, and location.

We built generalized linear models and used the ‘dredge’ function from package *MuMIn* (Barton, 2018) to perform model selection. We assessed the weight of each model using the delta AICc values (∆*i*; models were considered significant if the delta AICc value was <2 and the AIC weight close to 1; Burnham & Anderson, 2002; Symonds & Moussalli, 2011). Model averaging was performed when no single top model could be identified (i.e., Multiple top models with a ∆AICc value < 2).

## 3 RESULTS

We found a wide range of doubling times for introduced ungulate populations, from nearly exponential growth (with a difference in doubling time of 0.07 years between the theoretical and empirical curves) to large differences (up to 22.54 years) between empirical and exponential growth (Table 2).

For 17 populations, the slope calculated for the empirical populations was significantly less than that calculated for theoretical populations, that is, the slope calculated for the theoretical model fell above the 95% confidence intervals for the slope of the empirical curves (i.e., theoretical populations grew faster than real ones, asterisked in Table 2). Therefore, in 17/36 (47%) of ungulate populations, growth lagged significantly. None of the factors that we investigated contributed significantly to explain why populations lagged (*n* = 36, Table 4) or the degree of lagging population growth of introduced ungulates (*n* = 17, Table 5).

**TABLE 4:** Model averaging results from generalized linear models (GLM) indicating top variables from model selection for factors that affect introduced ungulate population growth (binary response of prolonged (1) or inherent (0) lagging populations) with variables from Table 3

	Estimate	*SE*	Adjusted *SE*	*z* value	2.50%	97.50%	*p* value
(Intercept)	−3.41	7.53	7.66	0.45	−18.42	11.61	0.656
Competition	0.67	0.85	0.87	0.77	−0.14	2.86	0.442
Region	0.66	0.98	1.00	0.67	−0.41	3.45	0.505
Gestation	1.20	3.23	3.29	0.36	−3.78	15.92	0.716
Predators	0.21	0.56	0.56	0.38	−0.44	2.70	0.704

**TABLE 5:** Model averaging results from generalized linear mixed‐effect models (GLMM's) indicating top variables from model selection for factors that affect introduced ungulate population growth (difference between observed and exponential doubling time) of lagging populations with variables from Table 3

	Estimate	*SE*	Adjusted *SE*	*z* value	2.50%	97.50%	*p* value
(Intercept)	8.22	30.75	34.38	0.24	−59.17	75.61	0.811
Competition	4.52	3.67	3.89	1.16	−0.81	12.59	0.245
Gestation	−1.68	11.94	13.41	0.13	−30.12	26.24	0.900
Dry season	−0.45	12.41	13.88	0.03	−29.58	28.55	0.974
Predators	−2.11	3.09	3.27	0.65	−11.39	1.93	0.518
Variance	0.81	2.19	2.35	0.34	−4.03	10.84	0.731
Hunting	−0.79	2.23	2.40	0.33	−11.18	4.48	0.743

## 4 DISCUSSION

Populations of introduced ungulates often grow exponentially upon release (Riney, 1964), which is a pattern we found in 53% of the populations. Of those species that did exhibit a lag, we could not identify any specific intrinsic or extrinsic variables that were significant contributors to delayed population growth. Our results are consistent with other studies that examined causes for lag phases in population growth in birds and plants and found no single cause of slow growth (Aagaard & Lockwood, 2014; Larkin, 2012).

This study is the first to systematically identify and analyze causes of population growth lags in mammals, specifically in introduced ungulates. Large mammals, such as ungulates, have been widely introduced to environments around the world and caused various environmental problems (Cote et al., 2004; Hernandez et al., 2018; Riney, 1964). Sika deer (*Cervus nippon*) and muntjac (*Muntiacus reevesi*) are listed in the top 10 worst alien species for Europe when ranked by impact, while chital deer (*Axis axis*), aoudad (*Ammotragus lervia*), white‐tailed deer (*Odocoileus virginianus*), and mouflon (*Ovis gmelini*) rank within the top 100 (Nentwig et al., 2018). We found that four of these five species (muntjac were not included in this study) displayed lag phases in parts of their introduced ranges. We thus conclude that many populations of introduced ungulates currently persisting at low numbers may represent significant economic and environmental threats that are yet to be recognized.

Investigations into causes of lag phases in plants and birds show that they are idiosyncratic and that they are not predictable using a given set of explanatory variables, and our results are consistent with these studies (Larkin, 2012; Aagaard & Lockwood, 2014; Mack et al., 2000). The factors that allow introduced species to break out of lag phases are variable among species and populations (Aagaard & Lockwood, 2014). Environmental change may trigger species that are lagging to suddenly grow and spread rapidly (Crooks & Soule, 1999; Fisher et al., 2020; Fujisaki et al., 2010; Rilov et al., 2004; Romagosa & Labisky, 2000), as they (through climate and/or anthropogenic influences) enable accelerated expansion into previously unoccupied areas (Fisher et al., 2020; Hengeveld, 1989; Witte et al., 2010).

One limitation of this study is that very few publications reported multiple population density estimates over time. Only by examining longitudinal data, we can distinguish more fine‐scale patterns of population growth. For example, a species that grows slowly may have a growth curve (as defined in this study) that appears similar to that of a species that grows quickly, but experiences frequent population crashes. For these reasons, it would be ideal to have detailed longitudinal datasets of introduced ungulate population densities to use for this study. On the other hand, we found that our estimates of population growth patterns were fairly good for species with available estimates of population size at multiple points, so we feel our estimates were at least partially representative of likely population growth trajectories for many of these species. Another limitation of our study is that several factors, such as parasite load, and the extent of hunting pressure, were not available in the literature (Albrecht et al., 2009; Carey & McLean, 1983; Kock et al., 2010; Wade, 2007). While hunting was included in the analyses, we can only report on whether a species was hunted or not, and not the degree of hunting (particularly from private hunters). Removal of adult animals by hunters can significantly reduce both population size and growth rate (Festa‐Bianchet, 2003). Several of the species in this study likely experienced some level of hunting pressure, given that many of these populations were introduced specifically for the purpose of recreational hunting (*n* = 28), whereas others were subsequently hunted as trophies (Bender et al., 2019; Bradshaw & Brook, 2007; Fuller et al., 2018). If unmeasured factors, such as degree of hunting, predation rates, and population crashes, significantly influenced population growth in much of our dataset, these factors should all lead to longer lag phases. Given that our study still showed species that exhibited exponential growth, or inherent lags, with this population bias, we believe our growth models are an adequate reflection of biological reality. A final limitation is that there may have been methodological differences in the collection of abundance data. While these differences could not be quantified, these populations still provide valuable information on ungulate population growth, provided that the limitations of using these data are recognized.

Since the eradication and containment of introduced species is best done when populations are small, it is important to identify early which species may be in inherent or prolonged lag phases (Buhle et al., 2005; Simberloff, 2003). Species introduced to new environments may be present in low numbers now and seem under control for many years, but still have the potential for explosive growth in the future. It is cases such as these in which managers need to be cognizant of the potential for these populations to suddenly increase. With a better understanding of the factors causing prolonged lags, rapidly accelerated population growth can be anticipated, and pre‐emptive controls put in place (Fagan et al., 2002). More work is needed to predict how invasive ungulate populations may grow in the future.

## CONFLICT OF INTEREST

None.

## AUTHOR CONTRIBUTIONS

**Catherine L. Kelly:** Conceptualization (equal); data curation (lead); formal analysis (lead); investigation (equal); methodology (equal); writing – original draft (equal); writing – review and editing (equal). **Lin Schwarzkopf:** Conceptualization (equal); formal analysis (equal); methodology (equal); supervision (equal); writing – review and editing (equal). **Iain J. Gordon:** Conceptualization (equal); methodology (equal); supervision (equal); writing – review and editing (equal). **Ben Hirsch:** Conceptualization (equal); formal analysis (equal); methodology (equal); supervision (equal); writing – review and editing (equal).

## Supporting information

Figure S1‐S7

Table S1‐S4

## Data Availability

Sources for all data are listed in the reference lists and are publicly available in the cited literature and databases.
