# Supplementary material for: Population growth lags in introduced species
Source: Ecol Evol. 2021 Mar 9;11(9):4577–87. doi: 10.1002/ece3.7352 (PMC8093750; doi:10.1002/ece3.7352)
Supplement: Supplementary file 1 — Figure S1‐S7 [file ECE3-11-4577-s001.docx]

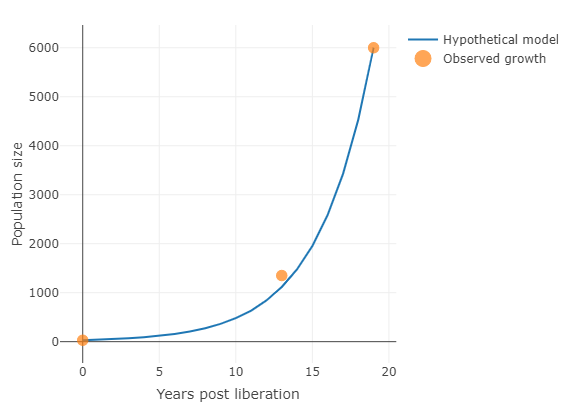


Figure 1. Observed population size (orange points) plotted against hypothetical curve (blue line) for *Rangifer tarandus* introduced to St Matthew Island (Alaska; Klein 1968)


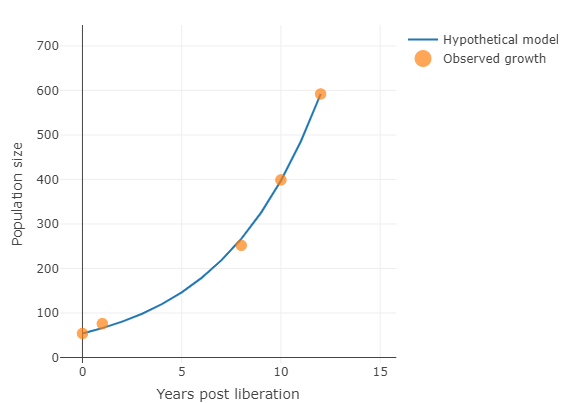


Figure 2. Observed population size (orange points) plotted against hypothetical curve (blue line) for *Cervus nippon* self-introduced to Japan (Kaji et al. 2004)


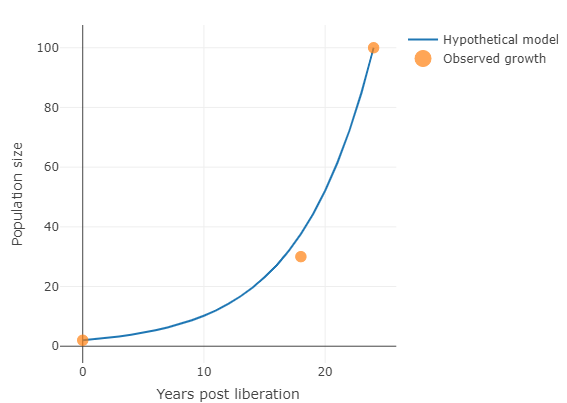


Figure 3. Observed population size (orange points) plotted against hypothetical curve (blue line) for *Cervus unicolor* self-introduced to New Zealand (Thomson 1922)


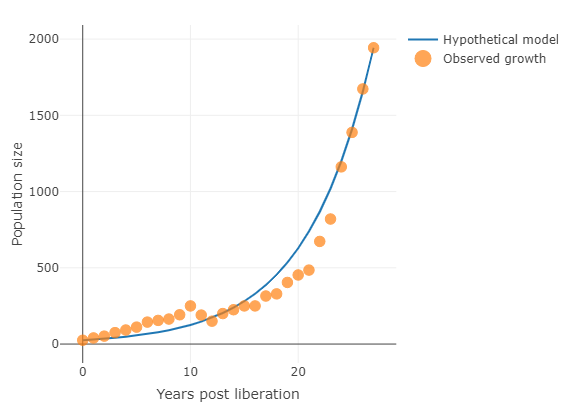


Figure 4. Observed population size (orange points) plotted against hypothetical curve (blue line) for *Rangifer tarandus* introduced to St Paul Island (Alaska; Scheffer 1951)


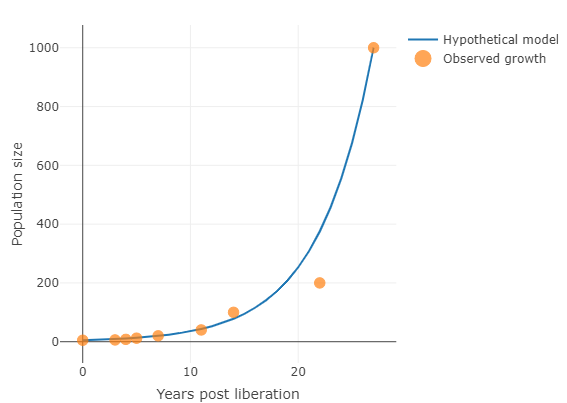


Figure 5. Observed population size (orange points) plotted against hypothetical curve (blue line) for *Odocoileus virginianus* introduced to Finland (Kekkonen et al. 2012)


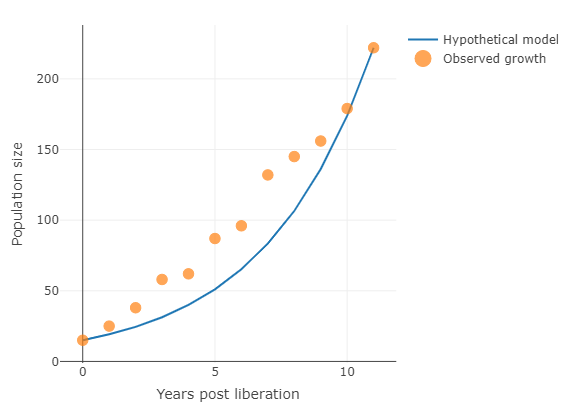


Figure 6. Observed population size (orange points) plotted against hypothetical curve (blue line) for *Rangifer tarandus* introduced to St George Island (Alaska; Scheffer 1951)


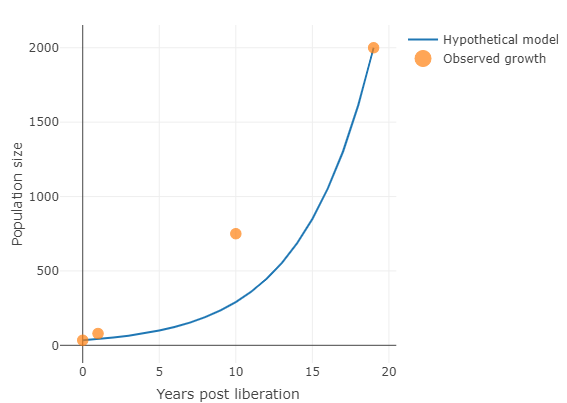


Figure 7. Observed population size (orange points) plotted against hypothetical curve (blue line) for *Ammotragus lervia* introduced to Spain (Cassinello et al. 2004)


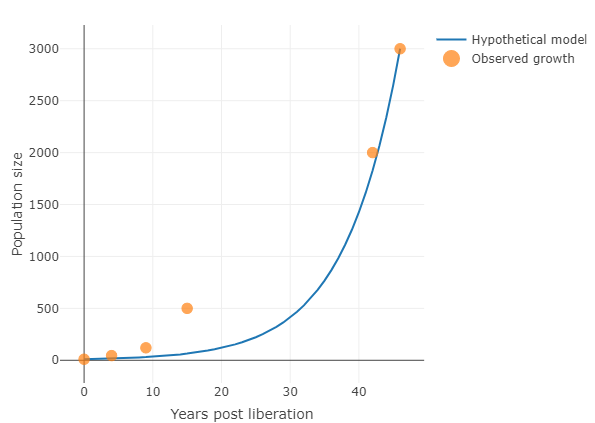


Figure 8. Observed population size (orange points) plotted against hypothetical curve (blue line) for *Rangifer Tarandus* introduced to South Georgia Island (Leader-Williams 1980)
