# Supplementary material for: Population growth lags in introduced species
Source: Ecol Evol. 2021 Mar 9;11(9):4577–87. doi: 10.1002/ece3.7352 (PMC8093750; doi:10.1002/ece3.7352)
Supplement: Supplementary file 2 — Table S1‐S4 [file ECE3-11-4577-s002.docx]

Table 1. Slopes of introduced ungulates compared with exponential population models, sorted in descending order from lowest to highest difference in slope.

Sources: (1) Klein (1968); (2) Chapui et al. (1994); (3) Scheffer (1951); (4) Tomich (1969); (5) Dvorak & Catalano (2016); (6) Colchero et al. (2009); (7) Steinbach et al. (2018); (8) Kaji et al. (2004); (9) Webley et al. (2004); (10) Thomson (1922); (11) Kekkonen et al. (2012); (12) Cassinello et al. (2004); (13) Cassinello (1998); (14) Judge et al. (2017); (15) Gogan et al. (2001); (16) Nogales et al. (2006); (17) Franklin & Grigione (2005); (18) Bender et al. (2019); (19) Leader-Williams (1980); (20) Barrau & Devambez (1957); (21) McCullough et al. (2009); (22) Caughley (1970); (23) Tustin & Challies (1978); (24) Bentley (1967); (25) Brennan & Pople (2016); (26) Graf & Nichols (1966); (27) Flesch et al. (2016); (28) Boulton & Freeland (1991); (29) Saalfeld & Edwards (2010); (30) Fuller et al. (2018); (31) Cooke (2009); (32) Bradshaw & Brook (2007); (33) Kopij (2017)

** species for which the theoretical exponential growth rate was significantly less than the 95% confidence intervals of empirical exponential growth models.

| Species (source) | Location | Introduced environment | Observed slope | Exponential slope | Difference |
| --- | --- | --- | --- | --- | --- |
| *Cervus unicolor* (10) | New Zealand | non-tropical | 0.24 | 0.25 | 0.01 |
| *Rangifer tarandus* (St Matthew) (1) | Alaska | non-tropical | 0.28 | 0.30 | 0.02 |
| *Ammotragus lervia* (12) | Spain | non-tropical | 0.21 | 0.24 | 0.03 |
| *Cervus timorensis* (9) | Australia | non-tropical | 0.23 | 0.26 | 0.03 |
| *Rangifer tarandus* (St George) (3) | Alaska | non-tropical | 0.25 | 0.28 | 0.04 |
| *Rangifer tarandus* (Haute) (2) | Kerguelen Isl. | non-tropical | 0.24 | 0.29 | 0.04 |
| *Ovis gmelini musimon* (2) | Kerguelen Isl. | non-tropical | 0.33 | 0.37 | 0.05 |
| *Cervus nippon* (8) | Japan | non-tropical | 0.20 | 0.25 | 0.05 |
| *Ovis gmelini* (14) | Hawaiꞌi | tropical | 0.18 | 0.25 | 0.07 |
| *Ovis Canadensis* (6) | Mexico | non-tropical | 0.21 | 0.28 | 0.07 |
| *Capreolus capreolis* (7) | Germany | non-tropical | 0.21 | 0.29 | 0.07 |
| *Ovis gmelini* (16) | Canary Isl. | non-tropical | 0.16 | 0.25 | 0.09 |
| *Ammotragus lervia* (16) | Canary Isl. | non-tropical | 0.15 | 0.25 | 0.10 |
| *Odocoileus virginianus* (11) | Finland | non-tropical | 0.20 | 0.30 | 0.11 |
| *Rangifer tarandus* (Grande-Terre) (2) | Kerguelen Isl. | non-tropical | 0.35 | 0.46 | 0.11 |
| *Odocoileus hemionus* (5) | USA | non-tropical | 0.27 | 0.38 | 0.11 |
| *Rangifer tarandus* (St Paul) (3) | Alaska | non-tropical | 0.16 | 0.28 | 0.11 |
| *Antilocapra americana* (4) | Hawaiꞌi | tropical | 0.27 | 0.41 | 0.14 |
| *Dama dama* (15) | USA | non-tropical | 0.15 | 0.29 | 0.14 |
| ***Rangifer tarandus* (19) **** | **South Georgia Isl.** | **non-tropical** | 0.12 | 0.29 | 0.17 |
| ***Rusa timorensis* (20) ****** | **New Caledonia** | **tropical** | 0.13 | 0.29 | 0.17 |
| ***Cervus nippon* (21) **** | **USA** | **non-tropical** | 0.10 | 0.27 | 0.17 |
| ***Oreamnos americanus* (27) ****** | **USA** | **non-tropical** | 0.06 | 0.23 | 0.17 |
| ***Hemitragus jemlahicus* (22,23) **** | **New Zealand** | **non-tropical** | 0.08 | 0.26 | 0.18 |
| ***Ammotragus lervia* (13) **** | **USA** | **non-tropical** | 0.18 | 0.36 | 0.19 |
| ***Camelus dromedarius* (29) **** | **Australia** | **tropical** | 0.06 | 0.25 | 0.19 |
| ***Bubalus bubalis* (28) **** | **Australia** | **tropical** | 0.06 | 0.25 | 0.19 |
| ***Axis axis* (15) **** | **USA** | **non-tropical** | 0.09 | 0.29 | 0.20 |
| ***Oryx gazella gazelle* (18) ****** | **USA** | **non-tropical** | 0.15 | 0.35 | 0.20 |
| ***Bos javanicus* (32) **** | **Australia** | **tropical** | 0.04 | 0.24 | 0.20 |
| ***Axis axis* (26) **** | **Hawaiꞌi** | **tropical** | 0.09 | 0.31 | 0.22 |
| ***Axis axis* (24,25) **** | **Australia** | **tropical** | 0.07 | 0.30 | 0.23 |
| ***Cervus nippon* (33) ****** | **Poland** | **non-tropical** | 0.03 | 0.29 | 0.26 |
| ***Lama guanicoe* (17) ****** | **Falkland Islands** | **non-tropical** | 0.17 | 0.46 | 0.29 |
| ***Odocoileus virginianus* (30) **** | **Canada** | **non-tropical** | 0.05 | 0.38 | 0.33 |
| ***Hydropotes inermis* (31) ****** | **England** | **non-tropical** | 0.06 | 0.46 | 0.40 |

Table 2. Growth rates (r) of introduced ungulates compared with exponential population models, sorted in descending order from lowest to highest difference in r.

** species for which the theoretical exponential growth rate was significantly less than the 95% confidence intervals of empirical exponential growth models.

| Species (source) | Location | Introduced environment | Observed r | Exponential r | Difference |
| --- | --- | --- | --- | --- | --- |
| *Rangifer tarandus* (St Matthew) (1) | Alaska | non-tropical | 1.32 | 1.33 | 0.01 |
| *Rangifer tarandus* (Grande-Terre) (2) | Kerguelen Isl. | non-tropical | 1.42 | 1.46 | 0.04 |
| *Rangifer tarandus* (St George) (3) | Alaska | non-tropical | 1.28 | 1.32 | 0.04 |
| *Ovis gmelini musimon* (2) | Kerguelen Isl. | non-tropical | 1.39 | 1.45 | 0.06 |
| *Rangifer tarandus* (Haute) (2) | Kerguelen Isl. | non-tropical | 1.28 | 1.33 | 0.06 |
| *Ammotragus lervia* (12) | Spain | non-tropical | 1.24 | 1.31 | 0.07 |
| *Ovis Canadensis* (6) | Mexico | non-tropical | 1.23 | 1.33 | 0.09 |
| *Capreolus capreolis* (7) | Germany | non-tropical | 1.24 | 1.33 | 0.09 |
| *Cervus unicolor* (10) | New Zealand | non-tropical | 1.18 | 1.27 | 0.09 |
| *Cervus nippon* (8) | Japan | non-tropical | 1.22 | 1.33 | 0.11 |
| *Antilocapra americana* (4) | Hawaiꞌi | tropical | 1.31 | 1.43 | 0.12 |
| *Odocoileus hemionus* (5) | USA | non-tropical | 1.30 | 1.46 | 0.16 |
| *Rangifer tarandus* (St Paul) (3) | Alaska | non-tropical | 1.17 | 1.34 | 0.16 |
| *Dama dama* (15) | USA | non-tropical | 1.16 | 1.34 | 0.18 |
| ***Rangifer tarandus* (19) **** | **South Georgia Isl.** | **non-tropical** | 1.13 | 1.34 | 0.21 |
| ***Rusa timorensis* (20) ****** | **New Caledonia** | **tropical** | 1.15 | 1.34 | 0.19 |
| ***Oreamnos americanus* (27) ****** | **USA** | **non-tropical** | 1.06 | 1.26 | 0.20 |
| ***Bubalus bubalis* (28) **** | **Australia** | **tropical** | 1.08 | 1.28 | 0.20 |
| ***Camelus dromedarius* (29) **** | **Australia** | **tropical** | 1.06 | 1.28 | 0.22 |
| ***Bos javanicus* (32) **** | **Australia** | **tropical** | 1.04 | 1.27 | 0.23 |
| *Cervus timorensis* (9) | Australia | non-tropical | 1.26 | 1.49 | 0.24 |
| ***Cervus nippon* (21) **** | **USA** | **non-tropical** | 1.10 | 1.35 | 0.24 |
| ***Axis axis* (15) **** | **USA** | **non-tropical** | 1.10 | 1.34 | 0.24 |
| *Odocoileus virginianus* (11) | Finland | non-tropical | 1.22 | 1.47 | 0.25 |
| ***Ammotragus lervia* (13) **** | **USA** | **non-tropical** | 1.19 | 1.44 | 0.25 |
| ***Oryx gazella gazelle* (18) ****** | **USA** | **non-tropical** | 1.16 | 1.42 | 0.26 |
| *Ovis gmelini* (14) | Hawaiꞌi | tropical | 1.20 | 1.47 | 0.27 |
| ***Axis axis* (24,25) **** | **Australia** | **tropical** | 1.07 | 1.34 | 0.27 |
| ***Axis axis* (26) **** | **Hawaiꞌi** | **tropical** | 1.07 | 1.35 | 0.28 |
| *Ovis gmelini* (16) | Canary Isl. | non-tropical | 1.18 | 1.46 | 0.29 |
| *Ammotragus lervia* (16) | Canary Isl. | non-tropical | 1.16 | 1.45 | 0.29 |
| ***Hemitragus jemlahicus* (22,23) **** | **New Zealand** | **non-tropical** | 1.06 | 1.35 | 0.29 |
| ***Cervus nippon* (33) ****** | **Poland** | **non-tropical** | 1.03 | 1.34 | 0.31 |
| ***Lama guanicoe* (17) ****** | **Falkland Islands** | **non-tropical** | 1.19 | 1.59 | 0.40 |
| ***Odocoileus virginianus* (30) **** | **Canada** | **non-tropical** | 1.06 | 1.47 | 0.41 |
| ***Hydropotes inermis* (31) ****** | **England** | **non-tropical** | 1.06 | 1.58 | 0.52 |

Table 3. AIC model selection top model results from generalized linear mixed effect models (GLMM’s) indicating top variables from model selection for factors that affect introduced ungulate population growth (difference between observed and exponential growth rates (r)) of lagging populations

| model | | Intercept | AICc | ∆i | weight | R^2^ |
| --- | --- | --- | --- | --- | --- | --- |
| **null** | **0.28** | **-24.00** | **0.00** | **0.67** | **0.89** |  |
| dry season | 0.35 | -20.30 | 3.64 | 0.11 | 0.87 |  |
| gestation | 0.64 | -19.80 | 4.17 | 0.08 | 0.90 |  |
| predators | 0.31 | -17.80 | 6.16 | 0.03 | 0.81 |  |
| variance | 0.46 | -17.60 | 6.36 | 0.03 | 0.94 |  |
| hunting | 0.24 | -17.50 | 6.43 | 0.03 | 0.90 |  |
| competition | 0.29 | -15.80 | 8.16 | 0.01 | 0.88 |  |
| gestation + dry season | 0.79 | -15.70 | 8.27 | 0.01 | 0.87 |  |
| hunting + dry season | 0.31 | -14.00 | 9.93 | 0.01 | 0.86 |  |
| gestation + variance | 1.11 | -13.60 | 10.34 | 0.00 | 0.95 |  |

Table 4. AIC model selection top model results from generalized linear mixed effect models (GLMM’s) indicating top variables from model selection for factors that affect introduced ungulate population growth (difference between observed and exponential slopes) of lagging populations

| model | (Int) | AICc | ∆i | weight | R^2^ |
| --- | --- | --- | --- | --- | --- |
| **null** | **0.23** | **-26.50** | **0.00** | **0.73** | **0.74** |
| dry season | 0.26 | -21.80 | 4.68 | 0.07 | 0.71 |
| gestation | 0.22 | -21.60 | 4.93 | 0.06 | 0.76 |
| predators | 0.26 | -21.00 | 5.52 | 0.05 | 0.65 |
| hunting | 0.19 | -19.80 | 6.74 | 0.03 | 0.76 |
| variance | 0.38 | -19.70 | 6.80 | 0.03 | 0.81 |
| competition | 0.24 | -18.30 | 8.26 | 0.01 | 0.69 |
| gestation + dry season | 0.29 | -16.30 | 10.21 | 0.00 | 0.73 |
| gestation + predators | 0.13 | -15.30 | 11.18 | 0.00 | 0.67 |
| dry season + predators | 0.27 | -15.30 | 11.22 | 0.00 | 0.65 |
